# Treatment of Tropical Dysentery by Enemeta of Tepid Water

**Published:** 1850-03

**Authors:** 


### ECLECTIC DEPARTMENT,

### AND SPIRIT OF THE MEDICAL PERIODICAL PRESS.

---

*Treatment of Tropical Dysentery by Enemeta of Tepid Water.* By E. Hare, Esq., 7th Bengal Irregular Cavalry.

Mr. Hare states, that having followed the mode of treatment here described, with marked success for five years, he was induced to make it known to his medical brethren in India, and has found that the results of their experience in its use are confirmatory of his own. He says:

Dr. Annesley's description of dysentery is as follows:

"The view we have given of disorders, which depend on accumulations of morbid secretions and local matters in the cavity of the larger bowels, shows in a remarkable manner, one of the very earliest stages of dysentery. Collections of excrementitious matters tend very evidently to irritate and inflame the mucous surface on which they lodge, and cause ulceration and even sphacelation in a very short period if neglected, or injudiciously treated. In a great many cases, dysentery is preceded by costive bowels, often of long duration. In a few instances, when the evacuations are copious, the diarrhœa subsides, and the patient escapes, at least for that time, a true dysenteric attack. This result seems to arise from the irritation produced in the bowels by the fæcal accumulations having subsided, in consequence of the irritating matters having been removed. Frequently the dysenteric symptoms are present from the first, the stools being scanty and streaked with blood, and with abdominal pain and tenesmus. In cases of this nature, the increased action of the muscular coats of the bowels, to which may be added the swollen state of the mucous membrane, especially about the sigmoid flexure and rectum, prevents the passage of the fæcal collections through their canal, and in many cases occasions complete obstruction, little passing away, but the perfect fluid secretions. In cases of this description, if the disease be not early subdued by very decided treatment, sloughing of the mucous coat take place, followed by involuntary motions, when the fæcal accumulations at last come away, such parts of them at least as have been dissolved, being washed off by the watery secretions poured from the irritated vessels of the inflamed surface."

This is the result of Dr. Annesley's large experience, and in most expressive language he shows the fatal effects of accumulations of fæces and acrid secretions in dysentery, and that the danger of this disease arises solely from their retention. This is corroborated by Dr. Gregory, Bampfield, and a host of others in equally large practice.

Mr. Hare says that every writer on the subject of dysentery with the exception only of Dr. Raleigh, is of the same opinion. He gives the following account of a case by Dr. Annesley:

"At first, purgatives brought away large fæeculent stools; but watery evacuations continuing, Dr. A. supposed that there was still lodgment of fæculent matter somewhere in the intestines, and that the watery evacuations were the effect of the irritation caused by its acrid qualities. Large doses of purgatives were therefore continued for several days, but no fæces came away. On his death, the small intestines were found loaded with fæeculent matter. The purgatives had brought copiously away at first the fæces from the large intestines, so that, before this evacuation, the man's abdomens was crammed with fæces, both large and small intestines."

Can there be a mistake here? And this was in India. But why multiply similar examples which may be found, not assertions, but *facts, cases*, in every writer on dysentery, from Galen till now. I cannot account for Dr. Raleigh's assertion, and it is vain to try; but I think, that where the accumulations are not very large, it is probable that nature herself, by throwing out fluid secretions, softens down and evacuates them; but in doing this, the gut is ulcerated and destroyed, though on dissection it is found empty. Dr. Annesley says:

The reason why scybala and solid fæces are seldom found in East Indian dysentery, is that the solid serous fluid copiously secreted from the irritated intestine washes it, squeezed, as it were, through the constricted canal in a liquid form."

These views, as to the retention of the excremental matter in dysentery, are elucidated and confirmed by the case of Dr. James Johnson, in Calcutta. Nothing relieved the symptoms till the calomel he took procured a copious *fæculent* bilious stool, succeeded, he says, "by such delightful feelings, that I prayed aloud." How then can Raleigh assert, that solid fæcal matters are *never* found in the acute dysentery of this country? I suspect, that in almost all that die, the efforts of nature have already softened and carried off in fluid the solid matters, and that Dr. R. would have got rid of these in the solid form, and saved often the lives of his patients, had he given purgatives more freely than he did; for he gives very small doses compared with others. If dysentery attack a man with costive bowels, (and one-third of the Europeans in this country have very costive bowels), there must be fæcal matter, and very often scybala in the intestines, which, from the nature of dysentery, cannot be expelled without nature's most painful efforts. I do not assert that dysentery is caused by accumulations; I think it rarely is. But when caused, the fœces it finds in the intestine, and the irritating secretions it afterwar ls adds, are the principal, perhaps *only* cause of its destructive termination, and I have no doubt that the danger of dysentery is essentially caused by the long contact of fæces and acrid secretions with the inflamed surface of the intestine; and if these be freely removed, the danger at once ceases.

No author except Raleigh says that solid fæces are never found; and any one that doubts that they are not almost always found, and in large quantity too, had better consult Annesley's plates of dysentery, which are not selected to illustrate my theory. He will find them *all*, and without exception, plates of fæcal accumulations, sometimes in large lumps here and there. Sometimes the intestine is so crammed, that the horizontal colon is bent by its own weight, down from the stomach to the region of the bladder.

I have shown that all writers on dysentery tried their best to empty the bowels by purgatives, as the one grand object in its treatment; for, this not accomplished, vain are their efforts to subdue the inflammation. Suppose an eye inflamed from cold or otherwise, put a lump of dysenteric fæces within the lid, and shut it up there, do you think that any bleeding, or twenty grain doses of calomel will prevent ulceration of the coats? This may be a coarse argument, but it is true, till it can be proved that one part of the great mucous membrane, continued in one piece from the eye to the anus, differs anywhere in its nature. What would you do in such a case? why, wash out the filth first, and then the inflammation will subside of itself, or with very moderate depletion.

The treatment advocated by Mr. Hare in acute dysentery, is, then, the injection of warm water by means of an elastic tube passed above the sigmoid flexure, as recommended some years ago, for other diseases as well as dysentery, by Dr. O'Beirne. Mr. Hare says:

By passing the elastic tube above the sigmoid flexure, you can inject from four to six pints, and thoroughly wash out the intestine from cœcum to anus.

It is satisfactorily proved in the work of Dr. O'Beirne, that the chief obstruction to the fæces always is in the sigmoid flexure, the rectum and itself remaining almost empty both in health and disease. The fæces accumulate, therefore, above the sigmoid flexure, the twistings of which, narrowed as it is in dysentery by spasm, and the swollen state of the mucous membrane, form so complete an obstruction, that nothing but fluid, and that with difficulty, can pass. The accumulation itself, too, greatly increases its own obstruction by impeding the circulation of the mucous membrane below it almost to strangulation, and causing sometimes so much swelling, that the mucous membrane protrudes through the anus, causing prolapsus. Now, Bampfield and Annesley, with their syringe, had no means of injecting more than the rectum, and a little, perhaps, of the sigmoic flexure; but they could never reach, through the obstructed sigmoid, the fæces beyond. The irritable rectum, too, on the slightest distension, contracts on and expels the injection. Even in health, if a small quantity of fæces passes into the rectum, it produces an instant desire to stool; but large quantities may accumulate for weeks above it without causing this sensation.

If, then, a tube can be passed directly into the fæcal mass, above the sigmoid flexure, this feeling, on injecting, will not be immediately felt, and the water, by the pumping action, being intimately mixed with the fæces, it is evident that the injection cannot return without fæcal matter with it. It is astonishing how readily a hard mass of fæces will soften and break down in water. Any one may prove this for himself without much trouble. The tube, too, can be moved up and down, and the water made to wash its way, at all points of the descending colon. By the first injection, some fæcal matter, at least, will be softened and removed, and the injection can be applied again and again, till the relieved intestine eject itself the harder matters, reduced, as they must be, in size, if any remain; and this, in fact, is the way in which purgatives evacuate the fæces in dysentery, having first reduced them to a soft or watery consistence by the fluid secretions they elicit from the intestines; but what increased irritation must they cause in doing this.

*Chronic Dysentery.*—If injections are valuable in acute, still more are they in chronic dysentery. This disease Annesley describes in the following terms:

"The inflammation of the acute stage leaves the villous coat of the colon throughout hypertrophied. Its papillæ having an erect rough appearance and feel, of a dark red or purplish hue, with here and there livid patches of congestion, particularly at the caput coli, and transverse arch, where ulcers ore often found. The sigmoid flexure is often very much contracted, its villous coat of unnatural thickness, and inflamed as well as the rectum. The lining membrane of the small bowels for some way up, is in much the same state, ash-colored ulcers, &c."

Now, I ask, is this a condition of the intestine likely to be improved by the irritation of purgatives, and yet the contraction of the colon, particularly the sigmoid flexure, together with the loss of muscular power and peristaltic action, necessarily succeeding the acute stage of the inflammation, render it absolutely necessary that they, or some substitute, should be given. The secretions, too, continue acrid, the appetite is often ravenous, while the powers of digestion are much weakened. The half-digested food and acrid secretions accumulate and distend the intestine above the contractions of the colon, causing dreadful irritation, and constant purging of fluid matters. Purgatives have, therefore, at all times been unwillingly resorted to, as a necessary evil; every writer inculcating in theory the use of the mildest laxatives, but in practice, in their *cases*, using from necessity calomel, epsom salts, black draughts, and compound jalap powder. To correct these, they used freely opiates and astringents, which, in checking the dangerous symptoms of the purging, produce accumulations. The accumulations require again purgatives; and so on the changes are rung, till the patient dies, or escapes only by the powers of a strong constitution; which, nevertheless, is often weakened, if not destroyed, by the previous treatment, for the acute stage.

See how dissatisfied Bampfield is under this destiny: "It must be granted that astringents display great powers over the morbidly secreting vessels; but it must also be admitted, that if they induce constipation, the morbid secretions are much increased, and it becomes necessary to abandon them. If the constipating effects of these astringents could be generally obviated, by the combination of a defined quantity of any purgative medicine, which would not decrease their astringent effects on the morbidly acting vessels, then it would appear, that the desideratum for the cure of this disease would be obtained."

Since 1822 Dr. O'Beirne has discovered this desideratum; yet no one has thought of making use of the method, though it is infinitely preferable to this half purgative, half astringent system, proposed here by Bampfield (if it were possible, which it is not). By the introduction of the elastic tube, you may wash out daily, with the most soothing applications, warm water, or milk and water, the excoriated intestine, removing its acrid secretions, and the fermenting, half-digested fæces, fomenting its tender surface the meanwhile, and softly stretching the strictured parts, with the gentle expansion of water. And when in the intestine, thus daily cleansed and soothed, the inflammation and irritation have been calmed, what numerous applications may be conveyed by the tube to the whole diseased surface of the colon, from the cœcum to the anus, gently to constringe the over-strained vessels, and heal the ulcers, the suggestion of the effect of these medicines, in disease of the mucous membrane of the eye, will be sufficient proof.

No constipation can be caused in this method of injecting, or even by giving these astringent medicines by the mouth, for the fæces ought to be daily removed by a large injection of water, previously to the exhibition of the small astringent injection, or astringent medicine by mouth, and great care ought to be taken, that a sufficiently large injection be daily thrown up to empty the bowels completely.

The proper quantity of injection is three or four pints, though Mr. Hare observes, the living intestine would hold a much larger quantity. Mr. H. proceeds to say:

The astringent injections I have found most useful, have been nitrate of silver, fifteen grains, dissolved in two and a half to three pints of water, a strong decoction of bark, and a mixture of catechu and chalk. The first and last I have found particularly useful. No doubt the nitrate of silver might be increased much in strength, but I have not been able yet to ascertain how much, from a dislike to try dangerous experiments without extreme caution. It is absolutely necessary to clear out the bowels first with a large injection, before injecting the medicinal one, which may be smaller.

I believe that inflammation of the mucous membrane of the colon ought to be treated on exactly the same principles as in the conjunctiva, where salivation is now rarely trusted to, but great use made of astringent lotions, with moderate depletion, and great attention to cleanliness, frequently washing away secretions by warm water or some mild lotion.

Free venesection will often be required in acute dysentery, and leeches in chronic, but very seldom, I think, mercury, though even this might be used as an ointment. It might be as well to observe, too, that it is by no means necessary, in all cases, to introduce the elastic tube far up the intestine; though really the inconvenience to the patient attending it, even in the worst cases, is so trifling as to make it almost a matter of indifference, if carefully done. After the first clearing of the intestine, a small distance, four or five inches, will suffice, and latterly, the common short rectum pipe, but the pump is indispensible; the common squirt will not do; and it is most requisite always to inject a full quantity, or we may fancy the intestine empty when it is not. A gentle mulling of the abdomen with the hand, while the injection is still in the bowels, assist it greatly in bringing away the accumulations. I generally keep the patient on his back, occasionally turning him to the left side. When he complains of, and I see that there is, distension of the abdomen, I cease to inject, and commence, if he can bear it, gentle mulling. The patient then sits up, and the injection never fails to carry all away with it.

The substance, then, of the whole argument is this. The long tube changes a huge internal abscess into an external, and enables us to wash out and cleanse from it its putrid contents. It also enables us to foment and soothe, by *local* applications, the sloughing and ulcerations these contents have caused on its surface. If any one can deny the utility of these means, I confess I cannot understand him. The consideration, too, that this abscess is in a vital organ, quickly prone to ulceration and sloughing, and, moreover, that its *function* is absorption, and, therefore, that it will rapidly absorb its own putrid contents, adds doubly to the force of the argument.

On those who may in future try this system, I would strongly urge, not to mix the old treatment with it, and dose their patients with calomel and purgatives which are directly opposed to it, and not to be led away by false theories of hydropathy and absorption of water, which are utterly without the shadow of probability, and to give the injections without limit in quantity but the patient's endurance. You cannot injure a piece of dead intestine by distending it with water, much less the living, which quickly contracts powerfully on its contents; and do not cease repeating the injection till you are satisfied that the colon is evacuated and cleansed.—*Edinburgh Med. and Surg. Jour.*

